# Contribution of Mass Spectrometry to the Advances in Risk Characterization of Marine Biotoxins: Towards the Characterization of Metabolites Implied in Human Intoxications

**DOI:** 10.3390/toxins15020103

**Published:** 2023-01-22

**Authors:** Pablo Estevez, Ana Gago-Martinez

**Affiliations:** Biomedical Research Center (CINBIO), Department of Analytical and Food Chemistry, Campus Universitario de Vigo, University of Vigo, 36310 Vigo, Spain

**Keywords:** marine biotoxins, LC–MS/MS, LC–HRMS, metabolism, biomarker, microsomes, hepatocytes, in vitro

## Abstract

A significant spread and prevalence of algal toxins and, in particular, marine biotoxins have been observed worldwide over the last decades. Marine biotoxins are natural contaminants produced during harmful algal blooms being accumulated in seafood, thus representing a threat to human health. Significant progress has been made in the last few years in the development of analytical methods able to evaluate and characterize the different toxic analogs involved in the contamination, Liquid Chromatography coupled to different detection modes, including Mass Spectrometry, the method of choice due to its potential for separation, identification, quantitation and even confirmation of the different above-mentioned analogs. Despite this, the risk characterization in humans is still limited, due to several reasons, including the lack of reference materials or even the limited access to biological samples from humans intoxicated during these toxic events and episodes, which hampered the advances in the evaluation of the metabolites responsible for the toxicity in humans. Mass Spectrometry has been proven to be a very powerful tool for confirmation, and in fact, it is playing an important role in the characterization of the new biotoxins analogs. The toxin metabolization in humans is still uncertain in most cases and needs further research in which the implementation of Mass Spectrometric methods is critical. This review is focused on compiling the most relevant information available regarding the metabolization of several marine biotoxins groups, which were identified using Mass Spectrometry after the in vitro exposition of these toxins to liver microsomes and hepatocytes. Information about the presence of metabolites in human samples, such as human urine after intoxication, which could also be used as potential biomarkers for diagnostic purposes, is also presented.

## 1. Introduction

In recent decades the proliferation of toxic phytoplankton has been representing a global risk affecting the marine environment and public health [1,2]. This phenomenon is known as harmful algal blooms (HABs). HABs occurrence is related to specific conditions not entirely understood and also to factors, such as changes in the temperature or the high presence of nutrients (e.g., phosphorous or nitrogen) [3,4]. Over the last decades, HABs have been more frequent and persistent all over the world. Climate change, eutrophication, globalization, invasive alien species migration, and/or the consequent increase in maritime transport and tourism can be some factors related to the increased incidence of HABs [5,6]. Marine biotoxins are secondary metabolites produced during HABs. Their role is not clearly identified as a possible protective system against predators or similar species [7]. They accumulate through the marine food chain, mainly in shellfish and fish, causing poisonings if these products are consumed by humans, aquatic organisms, or seabirds [8,9,10]. Marine biotoxins, originally classified as lipophilic and hydrophilic toxins based on their solubility, can also be classified based on their chemical structures. Different analogs of the same toxin class differing in slight structural differences cause the same poisoning, albeit a principal compound exhibiting most of the toxin potency, which is often the main analog responsible for the symptomatology [11]. Consequently, marine biotoxins causing specific poisoning are classified in the same group. Amnesic shellfish poisoning (ASP), paralytic shellfish poisoning (PSP), and pufferfish poisoning (PFP), whose toxins are not produced by HABs but by marine bacteria [12], are grouped in the hydrophilic marine biotoxins, while diarrhetic shellfish poisoning (DSP), yessotoxins (YTXs), azaspirazid poisoning (AZP), ciguatera poisoning (CP), neurotoxic shellfish poisoning (NSP), and cyclic imines (CIs) are included in the lipophilic marine biotoxins (Table 1). The European Union (EU) established regulatory limits for most of these marine biotoxins to reduce exposure to contaminated products and protect public health. On the other hand, the European Food Safety Authority (EFSA) published a series of scientific opinions on emerging marine biotoxins, such as CIs, CP, NSP, PFP, or palytoxins (PlTXs) [12,13,14,15].

Human poisonings related to the consumption of seafood contaminated with marine biotoxins have been reported worldwide [16,17]. Therefore, surveillance programs are being established to control the presence of HABs as well as marine biotoxins in seafood and the environment, and to protect consumers [18,19]. The detection methods used for monitoring these compounds are changing from non-specific approaches, such as the Mouse Bioassay (MBA), to more advanced and sophisticated technologies, such as Liquid Chromatography coupled to Mass Spectrometry (LC–MS) [20,21,22]. Despite the significant cost of LC–MS compared to MBA, these analytical techniques are being implemented in routine laboratories due to their sensitivity and specificity in identifying and quantifying specific toxins [23,24,25,26].

In spite of the increased knowledge on the different toxic profiles present worldwide in microalgae and seafood, the information related to the metabolization of the toxins in humans has been limited due to the lack of biological samples of poisoned humans and also of meals leftovers to study and compare the toxic profile. Therefore, this work aims to review the literature on mass spectrometry-based methods in the identification of marine biotoxins metabolites and their potential to be used as biomarkers in humans poisoned with marine biotoxins.

## 2. Mass Spectrometry to Identify Marine Biotoxins Metabolites

In the last years, there has been a tendency in the marine biotoxins monitoring field of using MS-based methodologies over other detection methods. This trend has been observed not only for the monitoring of regulated toxins, such as the use of HILIC–MS/MS instead of LC–FLD, for the monitoring of PSP toxins but also in the case of emerging marine biotoxins, such as CTXs or PbTxs [27,28,29,30]. Furthermore, the future perspectives on the monitoring of marine biotoxins would point towards the use of generic LC–MS methods based on High-Resolution Mass Spectrometry (HRMS) able to monitor multiple compounds in a single run [31]. Nevertheless, further advancements in MS technology, as well as in the availability of marine biotoxins reference materials, are needed to accomplish this objective [32].

The advantage of MS-based methods in the identification of marine biotoxin metabolites in biological samples relies on their ability to monitor the *m*/*z* of the putative biomarkers with superior sensitivity and specificity [33]. The additional performance of fragmentation experiments (MS/MS) and the comparison of the fragmentation pathways with the principal compound responsible for the toxicity in the seafood allows the identification of metabolites and potential biomarkers resulting from toxin metabolization.

However, apart from the difficulty of obtaining body fluids from humans poisoned with marine biotoxins, there are a series of limitations that hamper the application of MS-based methods in this field. First, the sensitivity of the LC–MS instruments for the monitoring of marine biotoxin metabolites cannot be evaluated due to the lack of reference materials of biomarkers to assess the appropriateness of the instrument parameters. Additionally, despite the efforts carried out in the preparation of standards and reference materials of marine biotoxins, some of these toxins are not commercially available (e.g., Caribbean ciguatoxins), therefore, limiting the performance of in vitro experiments to experimentally metabolize the toxins [34,35,36].

Therefore, while promising, the application of MS-based methods, such as targeted approaches using LC–MS/MS (Selected Reaction Monitoring, product, and precursor ion scan experiments) or untargeted analyses using LC–HRMS for the monitoring of marine biotoxins metabolites, is still in an early phase. However, there are a series of studies in which LC–MS was successfully applied in the identification of these metabolites [37,38]. In vitro studies, in which liver microsomes and hepatocytes are incubated with the toxins, allowed the identification of toxin metabolites by LC–MS. Liver microsomes and S9 fractions are tissues in which Cytochrome p450 (CYP) enzymes are abundantly expressed. These enzymes constitute a superfamily of different monooxygenases playing a role in the metabolization of drugs and toxins [39,40]. Biotransformation mechanisms, increasing compounds’ polarity and allowing their urinary or biliary excretion, are the main procedure for the elimination of toxic compounds. There are two different phases in the biotransformation of toxic xenobiotic compounds. Phase I introduces functionalization reactions such as oxidation, reduction, and hydrolysis [41]. On the other hand, phase II biotransformation involves conjugation with polar endogenous compounds, such as enzymes (e.g., sulfotransferases, glutathione S-transferases (GST) and UDP-glucuronosyltransferases (UGT)) [42].

The combination of in vitro exposure of liver microsomes or hepatocytes to the toxin with the LC–MS analysis of the metabolization products is the first step toward identifying metabolites of marine biotoxins that might be selected as biomarkers for diagnosis purposes. This approach has been applied to the following marine biotoxins.

### 2.1. Lipophilic Toxins Metabolites

The lipophilic marine biotoxins are the most widely studied toxins worldwide, and LC–MS methods are commonly used for monitoring their presence in seafood [20,25,43,44]. In the case of DSPs and AZAs, an internationally validated LC–MS/MS method has been established as the official method in the EU [45,46]. On the other hand, LC–MS has been used for the analyses of CIs, PbTxs, and CTXs; however, further research is needed to obtain reference materials of these compounds for the validation and harmonization of detection methods [47,48,49]. Accordingly, most of the research in the identification of lipophilic marine biotoxins metabolites has been focused on DSP and AZAs, while few studies were focused on CIs, PbTxs, and CTXs.

#### 2.1.1. Diarrhetic Shellfish Poisoning Toxins and Azaspirazids Metabolites

**Figure 1:**
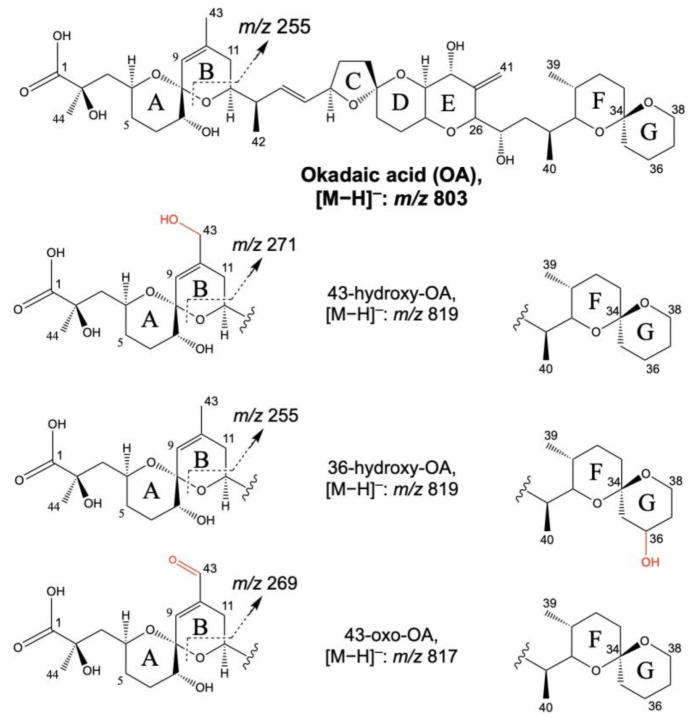
Structures of okadaic acid and its phase I metabolites determined by Liu et al. [60].

DSP toxins and AZAs are a group of marine biotoxins produced by dinoflagellates of the genus Dinophysis and Azadinium, respectively [50,51]. They are one of the most occurring toxins worldwide and, therefore, the most studied. Symptoms of DSP toxins include nausea, diarrhea, and vomiting. AZP cases show similar symptoms but also include a neurotoxic effect that can produce paralysis or respiratory distress [52,53,54,55]. Reference materials of these compounds are commercially available, allowing the development, optimization, and validation of LC–MS methodologies [56,57,58]. The identification of DSP toxins and AZAs metabolites in liver microsomes and hepatocytes incubated with the toxins was facilitated by the wide knowledge of the fragmentation pattern of these toxins during MS/MS experiments. For example, okadaic acid (OA) can be monitored in the negative ionization mode by selecting the deprotonated molecule *m*/*z* 803.5 [M−H]^−^ to the fragment *m*/*z* 255.1, as described in the EU-harmonized Standard Operating Procedure (SOP) for the determination of lipophilic marine biotoxins in mollusks by LC–MS/MS (Figure 1) [59,60]. Therefore, taking into account the expected phase I and phase II metabolizations and their mass shift (e.g., oxidation reactions: +16 Da), the metabolization products of OA can be monitored by the sensitive detection of these fragments in the Multiple Reaction Monitoring (MRM) modes. Most of the metabolites of this group of marine biotoxins were identified by the proposal of theoretical MRM transitions based on known and expected biotransformations. The combination of predicted MRM ion transitions with precursor and product ion scan analysis facilitated the obtention of structural information of the toxin metabolites [37].

Guo et al. [61] incubated OA with nine human recombinant cytochrome P450s, which represents 70% of cytochrome P450s present in the human liver and is involved in xenobiotic metabolism. CYP3A4 and CYP3A5 converted OA to a mixture of the same four metabolites. Three OA metabolites were detected at *m*/*z* 819 [M−H]^–^ and one at *m*/*z* 817 [M−H]^−^, and CYP3A4 showed a higher ratio of conversion with an overall yield of 31.3%, while it was 6.1% for the CYP3A5. These metabolites eluted before OA and were identified by LC–MS in full MS mode in the negative ionization mode. The chromatographic peaks corresponding to the metabolites were absent in the incubation mixture at 0 min and increased while incubated with the time. Three isobaric metabolites showed a prominent ion at *m*/*z* 819 [M−H]^−^ with a mass difference of 16 Da compared to OA (*m*/*z* 803 [M−H]^−^), indicative of a hydroxylation or epoxidation of the molecule. The fourth metabolite showed a prominent *m*/*z* 817 [M−H]^–^. MS/MS (MS^2^), and MS^3^ experiments and reactions of chemical interconversion allowed the identification of metabolites 2, 3, and 4 as 11S-hydroxy-OA, 11R-hydroxy-OA, and 11-oxo-OA, while the metabolite 1 was identified as a hydroxylated product with an unknown specific site. These metabolites were also detected in human liver microsomes incubated with OA. When compared with OA, they were slightly less potent inhibitors of serine–threonine protein phosphatase 2A (PP2A), seeming unlikely that these transformations detoxify OA. The structures of the OA metabolites previously identified by Guo et al. [61] were finally elucidated by Liu et al. [60] after the incubation of OA with human cytochrome P450 (CYP3A4). Two metabolites were determined by LC–MS/MS and 1D and 2D Nuclear Magnetic Resonance (NMR), while the third metabolite was identified by the oxidation reaction to a known metabolite. The structures of the metabolites initially identified as 11-hydroxy- and 11-oxo-OA were determined as 43-hydroxy- and 43-oxo-OA, while the metabolite with an unknown hydroxylation position was identified as 36-hydroxy-OA. All of these metabolites were OA-like inhibitors of PP2A (Figure 1 and Table 2).

A comparative study of the metabolite profiles after the incubation of OA in human and rat recombinant CYP enzymes was carried out by Kolrep et al. [62]. The authors observed different mechanisms of OA metabolism in the liver. CYP3A4 and CYP3A5 contribute to the detoxification of OA, showing a specific metabolite pattern. Despite the detection by LC–MS/MS in the Selected Reaction Monitoring (SRM) mode of the same hydroxylated metabolites, differing in +16 (+O), +14 (+O/−H_2_), and +32 (+2×O) Da from OA, human CYP3A4 detoxified a higher rate of OA compared to rat Cyp3a1 (Table 2). Therefore, the authors concluded that the transference of animal data on humans for risk assessment purposes should take into account the inter-species differences in the metabolism of OA. The hepatic metabolism of OA was also studied with S9 fractions from humans, rats, and rats with enzyme inducers in the absence or presence of NADPH [63]. LC–MS/MS was used to identify the metabolites produced during this experiment detecting metabolites differing in +16 (+O) and +14 (+O/−H_2_) Da with OA. A higher number of detoxifying metabolites were detected by LC–MS/MS in the S9 mix with NADPH-dependent enzymes of rats compared to the same enzymes in humans (Table 2) [63].

MS-based technologies were used by Kittler et al. [37] to elucidate phase I and phase II in vitro metabolites of lipophilic marine biotoxins using the S9 fraction of rat liver. The combination of LC–MS/MS for a first screening of the metabolites was followed by their confirmation by HRMS, allowing the identification of 47 metabolites from six toxins: OA, dinophysistoxins-1 and -2 (DTX1 and DTX2), yessotoxin (YTX), azaspiracid-1 (AZA1), and pectenotoxin-2 (PTX2) (Table 2). Product and precursor ion scanning experiments, as well as MS^3^ analyses, allowed identifying the carbon atom in which metabolization occurs or at least restricting it to a specific region of the structure. All the toxins studied in this work were converted into oxygenated phase I metabolites varying in the number of isomers. PTX2 and AZA1 showed metabolites with an addition of second and third oxygen, while only in AZA1 a loss of hydrogen was identified. Concerning phase II reactions, the authors only reported glucuronidation conjugates (+(C_6_H_8_O_6_)) for AZA1. The sensitivity offered by the targeted MRM mode of the triple quad instrument combined with the resolution of the HRMS instrument was demonstrated as a successful approach for unknown metabolite identification. The high mass resolution provided by LC–HRMS allowed the confirmation of metabolites initially identified by LC–MS/MS in the MRM mode.

Therefore, most of the metabolization reactions of these toxins, in both human and rat recombinant cytochrome, involve oxidation reactions to produce hydroxy- or oxo- metabolites. However, glucuronidation was detected only for AZA1.

#### 2.1.2. Spirolides Metabolites

Spirolides are a group of marine biotoxins of lipophilic nature produced by dinoflagellates of the genus Alexadrium, which belong to the cyclic imines (CIs) group [64,65,66]. CIs are characterized by having a unique seven-membered cyclic imine, as well as spiro-linked tricyclic ether groups [67,68]. The bioactive region of these compounds is the cyclic imine moiety, showing a neurotoxic effect through their interaction with nicotinic receptors of acetylcholine [69,70]. Despite being identified as toxic not only by intraperitoneal injection but also by oral administration [71], no food poisoning related to the consumption of shellfish containing CIs has been reported to date [13]. However, the study of their metabolism in humans is important to provide insights into their toxicity [38].

**Figure 2:**
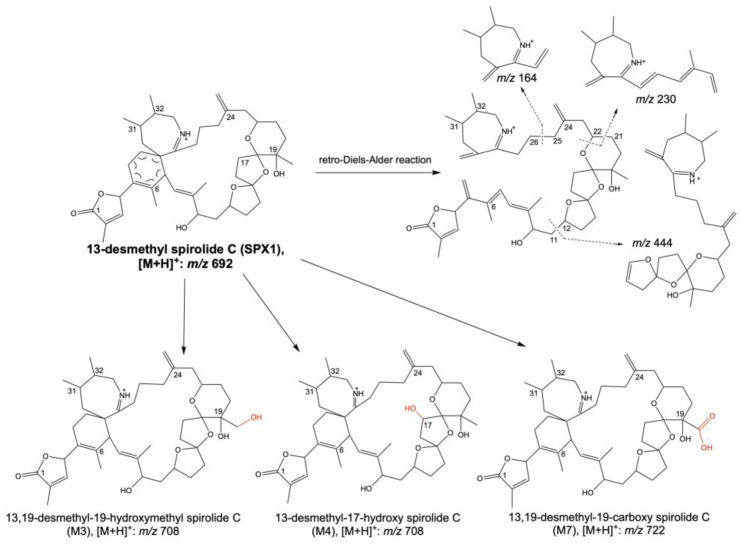
Fragmentation pathways of 13-desmethyl spirolide C and structures of the three phase-I in vitro metabolites structurally elucidated by Hui et al. [38]. Figure adapted from [72].

The combination of a first screening using diagnostic fragment ion with the accurate mass measurements provided by an Orbitrap MS instrument allowed the identification of nine in vitro metabolites of 13-desmethyl spirolide C (SPX1) [38]. Metabolites were generated in vitro using human liver microsomes exposed to SPX1. The identification of the metabolites was facilitated by the selection of a common fragment ion at *m*/*z* 164, characteristic of spirolides and containing the cyclic imine ring (Figure 2). The molecular formula of SPX1 metabolites was obtained by accurate mass measurements, and a total of nine phase-I metabolites of SPX1 were identified, including the following: hydroxylation, dihydroxylation, dehydrogenation, demethylation, and the oxidation of a quaternary methyl group to hydroxymethyl or carboxylic acid groups (Figure 2 and Table 3). Among the nine SPX1 metabolites, authors proposed the structures of three using MS/MS experiments: 13,19-didesmethyl-19-hydroxymethyl spirolide C (M3), 13-desmethyl-17-hydroxy spirolide C (M4), and 13,19-didesmethyl-19-carboxy spirolide C (M7) (Figure 2 and Table 3).

#### 2.1.3. Brevetoxins Metabolites

Brevetoxins (PbTxs) are a group of lipophilic marine biotoxins with a cyclic polyether structure produced by dinoflagellates of the genus Karenia [73]. PbTxs can accumulate in fish and shellfish, causing Neurotoxic Shellfish Poisoning (NSP) in humans. The symptoms of NSP include diarrhea, nausea, paresthesia, paralysis, and even coma [74]. NSP intoxications have been reported in the Gulf of Mexico, Florida (USA), and New Zealand, and more recently, PbTxs were detected in seafood from the EU waters [48,75,76]. PbTx-2 is the most abundant congener, while PbTx-1 is the most toxic.

**Figure 3:**
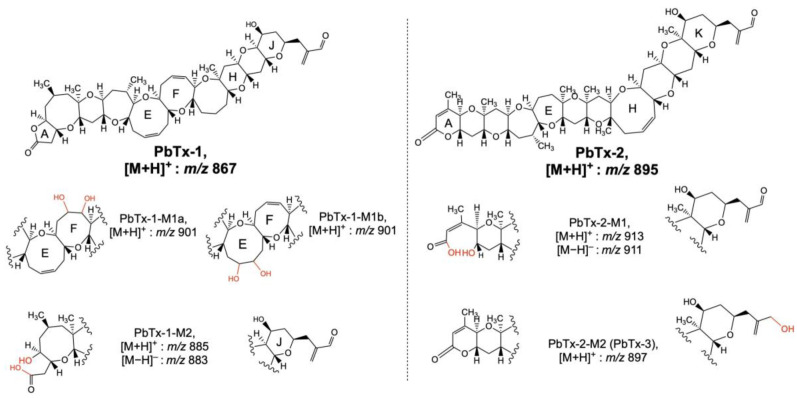
Proposed structures of brevetoxin metabolites by Wang et al. [77] after LC–MS/MS analyses of rat liver microsomes and hepatocytes incubated with PbTx-1 and PbTx-2.

The metabolism of PbTxs was studied by Wang et al. [77] by incubating PbTx-1 and -2 in rat liver hepatocytes and rat liver microsomes. Samples were analyzed by LC–MS in full scan and MS/MS mode after the removal of proteins using clean-up, showing that PbTx-1 was metabolized to two oxidized metabolites named PbTx-1-M1 and PbTx-1-M2 (Figure 3 and Table 4). LC–MS/MS results allowed to conclude that PbTx-1-M1 was formed by the conversion of the double bond of PbTx-1 E- or F- ring into a diol. On the other hand, PbTx-1-M2 resulted from the opening of the lactone A- ring of PbTx-1 followed by the addition of water. The metabolization products of PbTx-2 were also oxidized forms. A first metabolite named PbTx-2-M1 with a prominent ion detected at *m*/*z* 911 [M−H]^−^ whose fragmentation allowed to conclude that this compound is the hydrolysis product of PbTx-2 with a conversion of the lactone A-ring to a carboxylic acid and alcohol. The second metabolite was identified as PbTx-3 by comparison of the retention time and fragmentation pattern with the pure standard PbTx-3.

**Figure 4:**
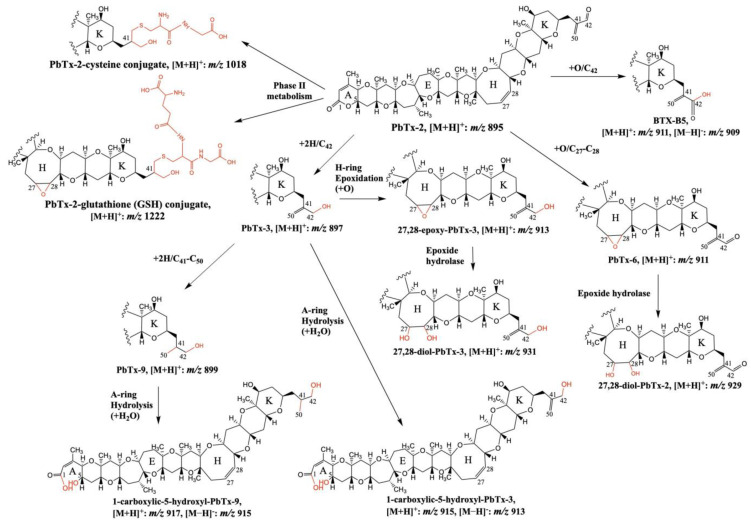
Proposed structures of phase I and II PbTx-2 metabolites detected after the in vitro incubation with rat liver microsomes and hepatocytes. Figure adapted from [78].

The in vitro metabolization of PbTx-2 was also evaluated by Radwan et al. [78]. PbTx-2 was incubated in the presence of an NADPH-generating system with rat liver cytochrome P450 enzymes. LC–MS/MS, in the full MS scan and product ion scan mode, was used to identify the metabolic products of PbTx-2 produced by CYP1A2 and CYP3A1. These CYP enzymes metabolized PbTx-2 to PbTx-3, being in agreement with the studies carried out by Wang et al. [77] (Figure 4 and Table 4). PbTX-9 ([M+H]^+^: *m*/*z* 899) and 27,28-diol-PbTx-2 ([M+H]^+^: *m*/*z* 929) were also identified by LC–MS/MS as PbTx-2 metabolic products, while CYP3A1 produced a significant amount of BTX-B5 ([M+H]^+^: *m*/*z* 911). The incubation of PbTx-2 with rat hepatocytes gave rise to phase I metabolites detected by LC–MS/MS with [M+H]^+^ at *m*/*z* 911, 913, 915, 917, and 931 showing that metabolizations occur in the A- and H- rings; epoxidations and hydrolysis, respectively [78] (Figure 4 and Table 4). Phase II metabolites of PbTx-2, glutathione ([M+H]^+^: *m*/*z* 1222) and cysteine ([M+H]^+^: *m*/*z* 1018) PbTx-2 conjugates, were also detected for the first time by LC–MS/MS and their structures were proposed based on the LC–MS data [78] (Figure 4).

Guo et al. [79] performed in vitro experiments incubating PbTx-2 with human liver microsomes. Three metabolites previously described, BTX-B5, PbTx-9, 41,43-dihydro-BTX-B5, and an additional unknown metabolite 41,43-dihydro-PbTx-2 were confirmed by LC–MS/MS (Table 4).

**Figure 5:**
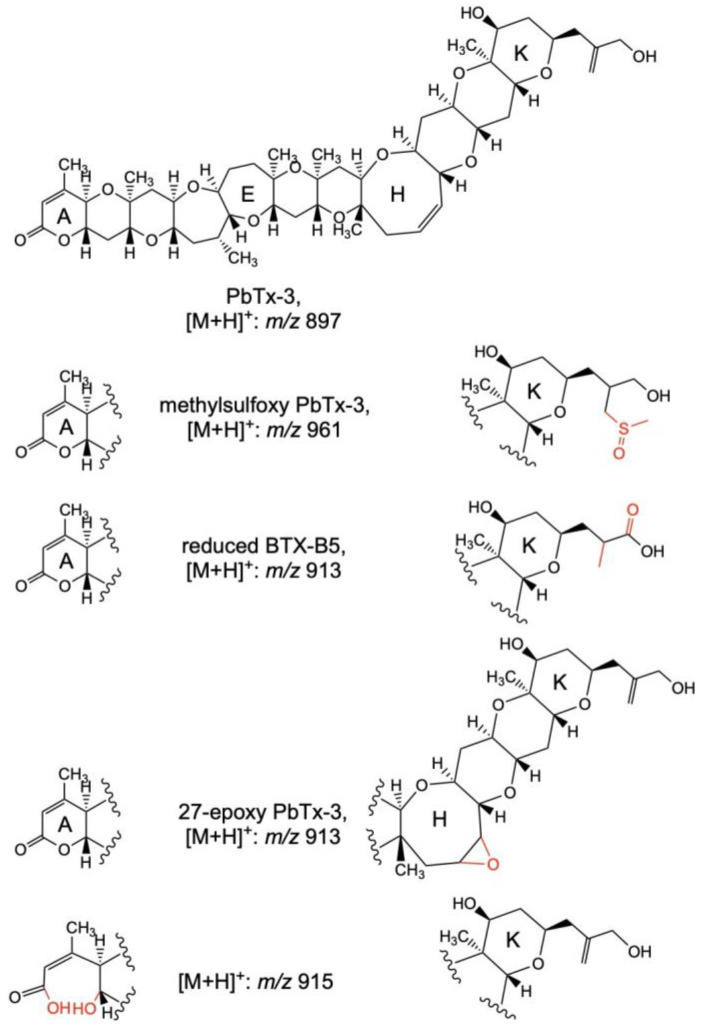
Proposed structures of brevetoxin biomarkers detected in urine samples of patients with NSP [80].

Biomarkers of PbTxs were first identified by Abraham et al. [80] in urine samples of patients diagnosed with NSP. Urine samples containing PbTxs were concentrated through C18 SPE and fractionated using LC. Fractions were analyzed by enzyme-linked immunosorbent assay (ELISA), and LC–MS/MS confirmed the presence of PbTxs in the active fractions. Most of the PbTxs metabolites identified in the urine samples differed from those present in both shellfish and shellfish meal remnants, being only PbTx-3 identified by LC–MS/MS in all the urine samples. A sensitive and selective LC–MS/MS method in the SRM mode was used to confirm the presence of PbTx-3 by monitoring the transition *m*/*z* 897 [M+H]^+^/725. The monitoring of this ion transition with two more resulting from water losses (*m*/*z* 897 [M+H]^+^/879 [M+H−H_2_O]^+^ and *m*/*z* 897 [M+H]^+^/861 [M+H−2H_2_O]^+^) and the comparison of the retention time with PbTx-3 reference material confirmed the presence of this compound in the urine samples. The composite PbTx level detected by ELISA correlated with the detected by LC–MS/MS for PbTX-3, being, therefore, a useful biomarker for the diagnoses of NSP. Among the other major urinary metabolites identified by LC–MS/MS were methylsulfoxy PbTx-3 ([M+H]^+^: *m*/*z* 961), a reduced BTX-B5 ([M+H]^+^: *m*/*z* 913), and 27-epoxy PbTx-3 ([M+H]^+^: *m*/*z* 913). Minor urinary PbTx metabolites were also detected at *m*/*z* 915 [M+H]^+^, *m*/*z* 901 [M+H]^+^, and *m*/*z* 887 [M+H]^+^ (Figure 5 and Table 5). An additional study carried out by Abraham et al. [81] followed the same approach of LC fractionation combined with the analysis of the fractions using ELISA to finally confirm by LC–MS/MS the major PbTxs metabolites present in the urine. PbTx-3 *m*/*z* 897 [M+H]^+^, an opened A-ring derivative of PbTx-3 *m*/*z* 915 [M+H]^+^, a reduced BTX-B5 *m*/*z* 913 [M+H]^+^, and 27-epoxy-PbTx-3 *m*/*z* 913 [M+H]^+^ were identified by LC–MS/MS, being in agreement with previous studies [80] (Figure 5 and Table 5).

#### 2.1.4. Ciguatoxins Metabolites

Ciguatoxins (CTXs) include a group of marine biotoxins produced by dinoflagellates of the genus Gambierdiscus and Fukuyoa [82,83]. CTXs are cyclic polyether’s around 1100 Da with a lipophilic nature and stable to temperature [84]. They accumulate in fish from tropical and subtropical areas causing Ciguatera Poisoning (CP) in humans. CP includes neurological, gastrointestinal, and cardiovascular symptoms, and there is no treatment or antidote [85,86]. Depending on the geographical region and structure, CTXs are classified as Pacific, Indian or Caribbean CTXs (P-CTXs, I-CTXs, and C-CTXs) [87,88,89].

**Figure 6:**
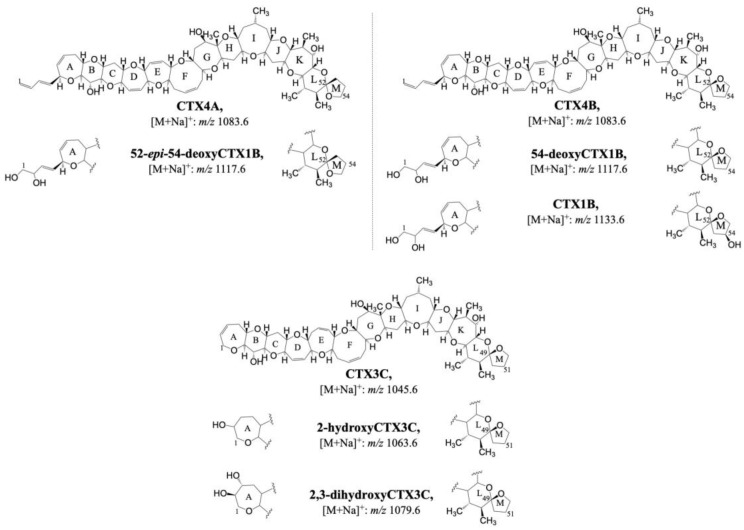
Proposed structures of phase I P-CTXs metabolites detected after the in vitro incubation with human CYP3A4, fish liver s9 fractions, and microsomal fractions from ciguateric and non-ciguateric fish.

The in vitro metabolism through the enzymatic oxidation of CTXs was first studied by Ikehara et al. [90]. Algal CTXs (CTX4A, CTX4B, and CTX3C) were exposed to human CYP3A4, fish liver S9 fractions, and microsomal fractions from ciguateric and non-ciguateric fish. CTX4A, CTX4B, and CTX3C were oxidized to CTX analogs typically detected in fish: CTX1B, 52-*epi*-54-deoxyCTX1B, 54-deoxyCTX1B, 2-hydroxyCTX3C, and 2,3-dihydroxyCTX3C (Figure 6 and Table 6). The reaction products were monitored by LC–MS/MS. The sensitive MRM mode monitoring CTXs [M+Na]^+^ as a precursor and product ion [91] and the comparison with the retention time of the reference materials allowed the confirmation of these metabolization reactions.

Phase-II metabolites of CTXs were recently detected by Gwinn et al. [92]. The in vitro incubation of C-CTX1 and C-CTX2 with liver microsomes of five fish species from the Caribbean Sea and also Atlantic Salmon showed the metabolization of these toxins to glucuronide products. Glucuronide conjugates (GlcA) were confirmed by HRMS/MS; however, the molecular site of GlcA attachment was not determined. C-CTX1 and C-CTX2 GlcA conjugates were detected in all the tested fish microsomes. On the other hand, GlcA conjugates of C-CTX1 and C-CTX2 were not detected after their incubation with both rat and human mammalian microsomes. Therefore, glucuronidation may be a specific metabolization of fish which might explain the sensitivity of humans after the exposition to C-CTXs [92].

### 2.2. Hydrophilic Toxins Metabolites

#### Paralytic Shellfish Toxins (PSTs) Metabolites

PSTs are a group of neurotoxic toxins produced dinoflagellates of the genus Alexandrium, Gymnodinium, and Pyrodinium that accumulate in seafood and can induce PSP in humans [93,94,95]. PSP symptoms include ataxia, respiratory depression or failure, tachycardia, and heart paralysis [96,97]. There is no treatment for this poisoning identified more than 30 analogs related to PSP [98].

**Figure 7:**
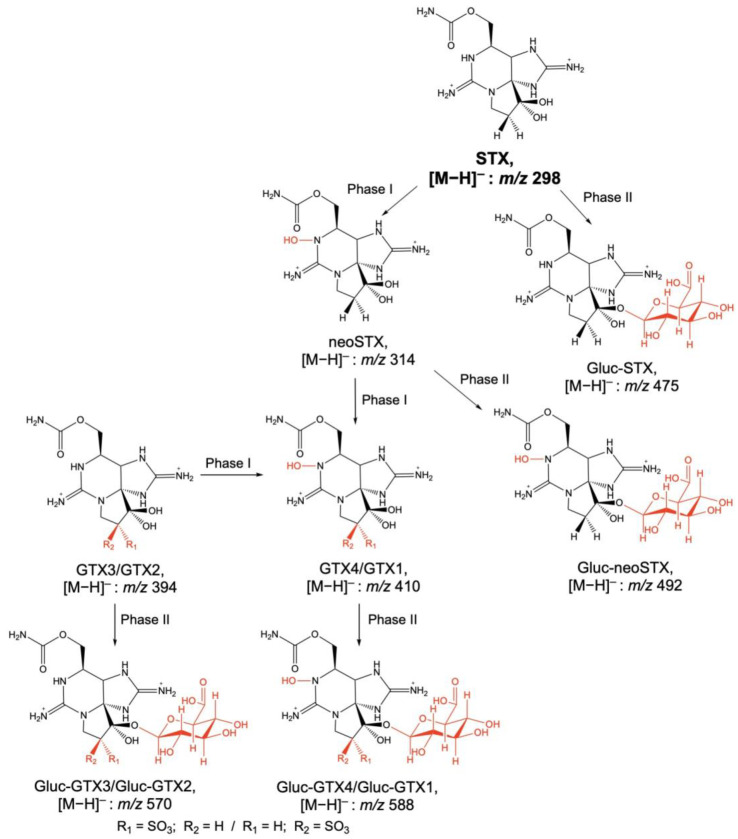
Oxidation (Phase I) and glucuronidation (Phase II) of PSTs after their in vitro incubation in human liver microsomes. Adapted from García et al. [99,100].

The phase I and II metabolizations of PSTs in humans were studied by the in vitro incubation of gonyautoxin-2 (GTX2) and gonyautoxin-3 (GTX3) in human liver microsomes [99]. García et al. [99] reported the oxidation and glucuronidation of GTX2 and GTX3 using LC–FLD and LC–MS. LC–MS analyses in the negative ionization mode confirmed the oxidation of GTX2 and GTX3 to gonyautoxin-1 (GTX1) and gonyautoxin-4 (GTX4) (Table 7 and Figure 7). Additionally, LC–FLD analyses showed evidence of in vitro glucuronidation of GTX2 and GTX3 being a possible route of metabolism and excretion of PSTs in humans. The previous work was completed by an additional study carried out by García et al. [100], in which neosaxitoxin (neoSTX), GTX2, GTX3, and saxitoxin (STX) were incubated in vitro with UDPGA/NADPH and human liver microsomes (Table 7 and Figure 7). Three metabolites of neoSTX, two of STX, and four of GTX2/3 were identified using HPLC–FLD and HPLC–ESI–IT–MS. Metabolites consisted of sequential oxidation and glucuronidation products being identified as the initial detoxification reactions for the elimination of PSTs in humans. Glucuronidation products were hydrolyzed using ß-glucuronidase, and 85% of the initial PSTs were metabolized at the end of the incubation. Gluc-GTX3/2 showed a major ion at *m*/*z* 570 corresponding to [M−H]^−,^ which gave rise to fragments at *m*/*z* 194.1, 322.6, and 395.2 being the ion at *m*/*z* 194.1 of the glucuronic ion. Gluc-GTX4/1 showed a similar pattern with a molecular ion at *m*/*z* 588.4 [M−H]^−^ and product ions at *m*/*z* 255.1, 323.3, and 403.3. Gluc-neoSTX was confirmed at *m*/*z* 492 [M−H]^−^ giving rise to fragments at *m*/*z* 226, 343, 379, 418, and 438. Finally, gluc-STX was detected at *m*/*z* 475 [M−H]^−^ being fragmented to *m*/*z* 163, 288, 316, 388, 410, and 418 [100].

## 3. Conclusions

The detection of marine biotoxin metabolites in human samples for diagnosis purposes is still in its early steps. Currently, most of the studies of metabolization are focused on in vitro studies combined with the MS detection of metabolization products. The availability of these data would facilitate the investigation of specific metabolites once samples from humans exposed to contaminations were available. While targeted approaches, such as LC–MS/MS in the MRM mode, allowed the identification of toxin metabolites, the future perspectives might be focused on untargeted approaches based on LC–HRMS combined with the use of software supporting and facilitating the LC–MS identification of marine biotoxin metabolites. In addition, the availability of reference materials of toxins with limited standards available, such as ciguatoxins, will be critical to carry out in vivo experiments to evaluate the metabolization process. The evolution of MS and the availability of sensitive MS approaches will also contribute to the identification, confirmation, and even characterization of the metabolites allowing the identification of potential biomarkers of food poisonings in human samples.

## Figures and Tables

**Table 1:** Main marine biotoxins in seafood produced by HABs and marine bacteria, the syndrome, and reference compounds.

Toxins	Syndrome	Parent Compound
Lipophilic	Diarrhetic Shellfish Poisoning (DSP)	Okadaic acid (OA)
	-	Yessotoxins (YTXs)
	Azaspiracid Poisoning (AZP)	Azaspiracids (AZAs)
	-	Cyclic Imines (CIs)
	Ciguatera Poisoning (CP)	Ciguatoxins (CTXs)
	Neurotoxic Shellfish Poisoning (NSP)	Brevetoxins (PbTxs)
Hydrophilic	Amnesic Shellfish Poisoning (ASP)	Domoic acid (DA)
	Paralytic Shellfish Poisoning (PSP)	Saxitoxin (STX)
	Pufferfish Poisoning (PFP)	Tetrodotoxin (TTX)

**Table 2:** Phase I and II in vitro metabolites of lipophilic marine biotoxins identified by LC–MS.

Toxin Molecular Formula	Q1/Q3 (*m*/*z*)	In Vitro Incubation		Metabolite	Q1/Q3 (*m*/*z*)	Reference
OA C_44_H_68_O_13_	[M−H]^−^ 803/255	Human	CYP3A4 and CYP3A5	+16 uma (+O): 36-hydroxy-OA,	[M−H]^−^ 819/255	[61]
				+16 uma (+O): 43-hydroxy-OA	[M−H]^−^ 819/271	
				+14 uma (+O/−H_2_): 43-oxo-OA	[M−H]^−^ 817/269	
			CYP3A4 and CYP3A5	+16 uma (+O): C_44_H_68_O_14_; M1, M2 and M3	[M−H]^−^ 819/255 [M−H]^−^ 819/255 [M−H]^−^ 819/271	[62]
				+14 uma (+O/–H_2_): C_44_H_66_O_14_; M4, M6	[M−H]^−^ 817/269 [M−H]^−^ 817/255	
			CYP3A5	+14 uma (+O/−H_2_): C_44_H_66_O_14_; M5	[M−H]^−^ 817/255	
				+32 uma (+2×O): C_44_H_68_O_15_; M7	[M−H]^−^ 835/255	
			S9 mix	+16 uma (+O): C_44_H_68_O_14_; M1, M2, M3	[M−H]^−^ 819/255 [M−H]^−^ 819/255 [M−H]^−^ 819/271	[63]
				+14 uma (+O/−H_2_): C_44_H_66_O_14_; M4	[M−H]^−^ 817/269	
		Rat	Cyp3a1	+16 uma (+O): C_44_H_68_O_14_; M1, M2 and M3	[M−H]^–^ 819/255 [M−H]^–^ 819/255 [M−H]^–^ 819/271	[62]
			CYP3A2	+16 uma (+O): C_44_H_68_O_14_; M3	[M−H]^–^ 819/271	
			S9 mix	+16 uma (+O): C_44_H_68_O_14_; M1, M2, M3	[M−H]^−^ 819/255 [M−H]^−^ 819/255 [M−H]^−^ 819/271	[63]
				+14 uma (+O/−H_2_): C_44_H_66_O_14_; M4	[M−H]^−^ 817/269	
				+16 uma (+O): C_44_H_68_O_14_	[M−H]^−^ 819/271 [M−H]^−^ 819/255	[37]
				+14 uma (+O/−H_2_): C_44_H_66_O_14_	[M−H]^−^ 817/269	
DTX1 C_45_H_70_O_13_	[M−H]^−^ 817/255	Rat	S9 mix	+16 uma (+O): C_45_H_70_O_14_	[M−H]^−^ 833/271 [M−H]^−^ 833/255	[37]
				+14 uma (+O/−H_2_): C_45_H_68_O_14_	[M−H]^−^ 831/269	
DTX2 C_44_H_68_O_13_	[M−H]^−^ 803/255			+16 uma (+O): C_44_H_68_O_14_	[M−H]^−^ 819/271 [M−H]^−^ 819/255	[37]
YTX C_55_H_82_O_21_S_2_	[M−H]^−^ 1141/106			+16 uma (+O): C_55_H_82_O_22_S_2_	[M−H]^−^ 1157/1077	[37]
AZA1 C_47_H_71_NO_12_	[M+H]^+^ 842/824			+176 uma (+C_6_H_8_O_6_): C_53_H_79_NO_18_	[M+H]^+^ 1018/848	[37]
				+16 uma (+O): C_47_H_71_NO_13_	[M+H]^+^ 858/824	
				+32 uma (2×O): C_47_H_71_NO_14_	[M+H]^+^ 874/856	
				+48 uma (3×O): C_47_H_71_NO_15_	[M+H]^+^ 890/872	
				−2 uma (−H_2_): C_47_H_69_NO_12_	[M+H]^+^ 840/822	
PTX2 C_47_H_70_O_14_	[M+NH_4_]^+^ 876/823			+16 uma (+O): C_47_H_70_O_15_	[M+NH_4_]^+^ 892/839	[37]
				+32 uma (2×O): C_47_H_70_O_16_	[M+NH_4_]^+^ 908/855	
				+48 uma (3×O): C_47_H_70_O_17_	[M+NH_4_]^+^ 924/871	

**Table 3:** Phase-I in vitro metabolites identified in human liver microsomes exposed to 13-desmethyl spirolide C. Data from Hui et al. [38].

Toxin Molecular Formula	*m*/*z* [M+H]^+^	Metabolite	*m*/*z* [M+H]^+^
SPX1 C_42_H_61_NO_7_	692.4521	+14 uma (+O/−H_2_): C_42_H_59_NO_8_; M1	706.4313
		+14 uma (+O/−H_2_): C_42_H_59_NO_8_; M2	706.4313
		+16 uma (+O): C_42_H_61_NO_8_; 13,19-desmethyl-19-hydroxymethyl spirolide C; M3	708.4470
		+16 uma (+O): C_42_H_61_NO_8_; 13-desmethyl-17-hydroxy spirolide C; M4	708.4470
		+18 uma (−CH_2_ + 2 × O): C_41_H_59_NO_9_; M5	710.4263
		+18 uma (−CH_2_ + 2 × O): C_41_H_59_NO_9_; M6	710.4263
		+30 uma (−2 × H + 2 × O): C_42_H_59_NO_9_; 13,19-desmethyl-19-carboxy spirolide C; M7	722.4263
		+32 uma (+2 × O): C_42_H_61_NO_9_; M8	724.4419
		+32 uma (+2 × O): C_42_H_61_NO_9_; M9	724.4419

**Table 4:** Phase-I in vitro metabolites of PbTx-1 and PbTx-2 incubated with rat liver hepatocytes and microsomes and identified by LC–MS/MS.

Toxin Molecular Formula	*m*/*z*	In Vitro Incubation	Metabolite	*m*/*z*	Reference
PbTx-1 C_49_H_70_O_13_	[M+H]^+^ 867	Rat liver hepatocytes	+34 uma; C_49_H_72_O_15_; PbTx-1-M1a/M1b	[M+H]^+^ 901	[77]
			+18 uma; C_49_H_72_O_14_; PbTx-1-M2	[M+H]^+^ 885 [M–H]^−^ 883	
PbTx-2 C_50_H_70_O_14_	[M+H]^+^ 895	Rat liver microsomes	+18 uma; C_50_H_72_O_15_; PbTx-2-M1	[M+H]^+^ 913 [M−H]^–^ 911	
			+2 uma; C_50_H_72_O_14_; PbTx-2-M2 (PbTx-3)	[M+H]^+^ 897	
		Rat CYP1A2 and CYP3A1	+2 uma; C_50_H_72_O_14_; PbTx-3	[M+H]^+^ 897	[78]
			+4 uma; C_50_H_74_O_14_; PbTX-9	[M+H]^+^ 899	
			+34 uma; C_50_H_72_O_16_; 27,28-diol-PbTx-2	[M+H]^+^ 929	
			+16 uma; C_50_H_70_O_15_; BTX-B5	[M+H]^+^ 911 [M−H]^−^ 909	
		Rat hepatocytes	+16 uma; C_50_H_70_O_15_; PbTx-6	[M+H]^+^ 911	
			+18 uma; C_50_H_72_O_15_; 27,28-epoxy-PbTx3	[M+H]^+^ 913	
			+20 uma; C_50_H_74_O_15_; 1-carboxylic-5-hydroxyl-PbTx-3	[M+H]^+^ 915 [M–H]^−^ 913	
			+22 uma; C_50_H_76_O_15_; 1-carboxylic-5-hydroxyl-PbTx-9	[M+H]^+^ 917 [M−H]^−^ 915	
			+36 uma; C_50_H_74_O_16_; 27,28-diol-PbTx-3	[M+H]^+^ 931	
		Human liver microsomes	+16 uma; C_50_H_70_O_15_; BTX-B5	[M+H]^+^ 911 [M–H]^−^ 909	[79]
			+4 uma; C_50_H_74_O_14_; PbTX-9	[M+H]^+^ 899	
			+18 uma; C_50_H_72_O_15_; 41,43-dihydro-BTX-B5	[M+H]^+^ 913	
			+2 uma; C_50_H_72_O_14;_ 41,43-dihydro-PbTx-2	[M+H]^+^ 897	

**Table 5:** PbTxs metabolites detected by LC–MS/MS in urine samples of patients suffering NSP.

Urine PbTxs Metabolite		*m*/*z*	References
Major metabolites	PbTx-3, C_50_H_72_O_14_	[M+H]^+^: *m*/*z* 897	[80,81]
	methylsulfoxy PbTx-3, C_51_H_76_O_15_S	[M+H]^+^: *m*/*z* 961	[80]
	reduced BTX-B5, C_50_H_72_O_15_	[M+H]^+^: *m*/*z* 913	[80,81]
	27-epoxy PbTx-3, C_50_H_72_O_15_	[M+H]^+^: *m*/*z* 913	[80,81]
Minor metabolites	opened A-ring derivative of PbTx-3, C_50_H_74_O_15_	[M+H]^+^: *m*/*z* 915	[80,81]
	C_49_H_72_O_15_	[M+H]^+^: *m*/*z* 901	[80]
	C_49_H_74_O_14_	[M+H]^+^: *m*/*z* 887	[80]

**Table 6:** Phase I in vitro metabolites of P-CTXs detected after the in vitro incubation with human CYP3A4, fish liver s9 fractions, and microsomal fractions from ciguateric and non-ciguateric fish.

Toxin Molecular Formula	Q1/Q3 (*m*/*z*)	Metabolite	Q1/Q3 (*m*/*z*)
CTX4A C_60_H_84_O_16_	[M+Na]^+^ 1083.6/1083.6	52-*epi*-54-deoxyCTX1B	[M+Na]^+^ 1117.6/1117.6
CTX4B C_60_H_84_O_16_	[M+Na]^+^ 1083.6/1083.6	54-deoxyCTX1B	[M+Na]^+^ 1117.6/1117.6
		CTX1B	[M+Na]^+^ 1133.6/1133.6
CTX3C C_57_H_82_O_16_	[M+Na]^+^ 1045.6/1045.6	2-hydroxyCTX3C	[M+Na]^+^ 1063.6/1063.6
		2,3-dihydroxyCTX3C	[M+Na]^+^ 1079.6/1079.6

**Table 7:** Phase I and II in vitro metabolites of paralytic shellfish toxins incubated in human liver microsomes [100].

Toxin Molecular Formula	Q1/Q3 (*m*/*z*)	Metabolite	Q1/Q3 (*m*/*z*)
STX C_10_H_17_N_7_O_4_	[M−H]^−^ 298/99 [M−H]^−^ 298/137 [M−H]^−^ 298/160	neoSTX	[M−H]^−^ 314/99 [M−H]^−^ 314/175 [M−H]^−^ 314/603 [M−H]^−^ 314/619 [M−H]^−^ 314/625
		Gluc-STX	[M−H]^−^ 475/163 [M−H]^−^ 475/288 [M−H]^−^ 475/316 [M−H]^−^ 475/388 [M−H]^−^ 475/410 [M−H]^−^ 475/418
neoSTX C_10_H_17_N_7_O_5_	[M−H]^−^ 314/99 [M−H]^−^ 314/175 [M−H]^−^ 314/603 [M−H]^−^ 314/619 [M−H]^−^ 314/625	GTX4/GTX1	[M−H]^−^ 410/210 [M−H]^−^ 410/254 [M−H]^−^ 410/323
		Gluc-neoSTX	[M−H]^−^ 492/226 [M−H]^−^ 492/342 [M−H]^−^ 492/379 [M−H]^−^ 492/418 [M−H]^−^ 492/438
GTX1 C_10_H_17_N_7_O_9_S	[M−H]^−^ 410/210 [M−H]^−^ 410/254 [M−H]^−^ 410/323	Gluc-GTX1	[M−H]^−^ 588/255 [M−H]^−^ 588/323 [M−H]^−^ 588/403
GTX2 C_10_H_17_N_7_O_8_S	[M−H]^−^ 394/297 [M−H]^−^ 394/311 [M−H]^−^ 394/365	GTX4/GTX1	[M−H]^−^ 410/210 [M−H]^−^ 410/254 [M−H]^−^ 410/323
		Gluc-GTX2	[M−H]^−^ 570/194 [M−H]^−^ 570/322 [M−H]^−^ 570/395
GTX3 C_10_H_17_N_7_O_8_S	[M−H]^−^ 394/297 [M−H]^−^ 394/311 [M−H]^−^ 394/365	GTX4/GTX1	[M−H]^−^ 410/210 [M−H]^−^ 410/254 [M−H]^−^ 410/323
		Gluc-GTX3	[M−H]^−^ 570/194 [M−H]^−^ 570/322 [M−H]^−^ 570/395
GTX4 C_10_H_17_N_7_O_9_S	[M−H]^−^ 410/210 [M−H]^−^ 410/254 [M−H]^−^ 410/323	Gluc-GTX4	[M−H]^−^ 588/255 [M−H]^−^ 588/323 [M−H]^−^ 588/403

## Data Availability

The data presented in this study are available in this article.
